# Use of Univent tube for intermittent lung isolation during thoracoscopic mediastinal tracheal resection and reconstruction: A case report: Erratum

**DOI:** 10.1097/MD.0000000000009622

**Published:** 2018-01-12

In the article, “Use of Univent tube for intermittent lung isolation during thoracoscopic mediastinal tracheal resection and reconstruction: A case report”,^[1]^ which appeared in Volume 96, Issue 50 of *Medicine*, Dr. Qian Li is the only corresponding author.
